# Persistence of Innate Immune Pathways in Late Stage Human Bacterial and Fungal Keratitis: Results from a Comparative Transcriptome Analysis

**DOI:** 10.3389/fcimb.2017.00193

**Published:** 2017-05-18

**Authors:** Jaya D. Chidambaram, Shichina Kannambath, Palepu Srikanthi, Manisha Shah, Prajna Lalitha, Shanmugam Elakkiya, Julien Bauer, Namperumalsamy V. Prajna, Martin J. Holland, Matthew J. Burton

**Affiliations:** ^1^Department of Clinical Research, London School of Hygiene and Tropical MedicineLondon, United Kingdom; ^2^Institute for Infection and Immunity, St. George's University of LondonLondon, United Kingdom; ^3^Cornea Department, Aravind Eye HospitalMadurai, India; ^4^Aravind Medical Research FoundationMadurai, India; ^5^Department of Pathology, University of CambridgeCambridge, United Kingdom

**Keywords:** keratitis, corneal ulcer, fungi, bacteria, transcriptome, fusarium, aspergillus, streptococcus

## Abstract

Microbial keratitis (MK) is a major cause of blindness worldwide. Despite adequate antimicrobial treatment, tissue damage can ensue. We compared the human corneal transcriptional profile in late stage MK to normal corneal tissue to identify pathways involved in pathogenesis. Total RNA from MK tissue and normal cadaver corneas was used to determine transcriptome profiles with Illumina HumanHT-12 v4 beadchips. We performed differential expression and network analysis of genes in bacterial keratitis (BK) and fungal keratitis (FK) compared with control (C) samples. Results were validated by RTqPCR for 45 genes in an independent series of 183 MK patients. For the microarray transcriptome analysis, 27 samples were used: 12 controls, 7 BK culture positive for *Streptococcus pneumoniae* (*n* = 6), *Pseudomonas aeruginosa* (*n* = 1), and 8 FK, culture positive for *Fusarium* sp. (*n* = 5), *Aspergillus* sp. (*n* = 2), or *Lasiodiplodia* sp. (*n* = 1). There were 185 unique differentially expressed genes in BK, 50 in FK, and 339 common to both [i.e., genes with fold-change (FC) < −4 or ≥4 and false discovery rate (FDR) adjusted *P* < 0.05]. *MMP9* had the highest FC in BK (91 FC, adj *p* = 3.64 E-12) and FK (FC 64, adj. *p* = 6.10 E-11), along with other MMPs (*MMP1, MMP7, MMP10, MMP12*), pro-inflammatory cytokines (*IL1B, TNF)*, and PRRs (*TLR2, TLR4)*. *HIF1A* and its induced genes were upregulated uniquely in BK. Immune/defense response and extracellular matrix terms were the most enriched Gene Ontology terms in both BK and FK. In the network analysis, chemokines were prominent for FK, and actin filament reorganization for BK. Microarray and RTqPCR results were highly correlated for the same samples tested with both assays, and with the larger RTqPCR series. In conclusion, we found a great deal of overlap in the gene expression profile of late stage BK and FK, however genes unique to fungal infection highlighted a corneal epithelial wound healing response and for bacterial infection the prominence of *HIF1A*-induced genes. These sets of genes may provide new targets for future research into therapeutic agents.

## Introduction

Microbial keratitis (MK) is a major cause of blindness worldwide, and affects an estimated 840,000 people per year in India alone (Whitcher et al., 2001). In severe corneal ulceration, despite adequate antimicrobial therapy, the disease can progress resulting in corneal perforation in up to 30% and loss of the eye in up to 25% of patients (Poole, 2002; Burton et al., 2011). The spectrum of organisms that cause MK varies geographically, with filamentous fungi (e.g., *Fusarium* sp. and *Aspergillus* sp.) accounting for up to 50% of all MK in tropical regions such as South India (Srinivasan et al., 1997). In more temperate climates, bacterial pathogens such as *Pseudomonas aeruginosa* and *Streptococcus pneumoniae* predominate, although there have been some notable outbreaks of filamentary fungal infections related to contact lens solutions in recent years (Tuft and Matheson, 2000; Galarreta et al., 2007; Gorscak et al., 2007).

Although some pathogens are able to produce enzymes that can damage the cornea, much of the corneal destruction in MK is likely due to an excessive host inflammatory response (Steuhl et al., 1987; Gopinathan et al., 2001). Several factors are thought to contribute to this tissue destruction and subsequent poor clinical outcome in MK. Tissue macrophages resident in the cornea detect pathogen-associated molecular patterns (PAMPs) via pattern recognition receptors (PRRs) such as Dectin-1 and toll-like receptors resulting in production of cytokines such as IL1B and chemokines (e.g., CXCL1, CXCL5) which result in a rapid influx of neutrophils (Leal et al., 2010; Sun et al., 2010). Activated neutrophils attempt to destroy the pathogen through production of reactive oxygen species and release of enzymes such as matrix metalloproteinases (MMPs) but these mechanisms can also damage surrounding host tissue. Additional sources of MMPs during MK include host corneal epithelial cells and activated keratocytes, causing an excess of these enzymes that can contribute to further tissue destruction, and even cornea perforation (Matsubara et al., 1991). By using a transcriptomic approach, Huang et al. investigated the entire murine corneal response to early *P. aeruginosa* infection and found increased gene expression for pro-inflammatory cytokines (e.g., *IL1B, TNF*), chemokines (e.g., *CXCL2*), in the corneas that developed perforation (Huang and Hazlett, 2003). Genes that protected against apoptosis, e.g., *BCL2*, were also upregulated in perforated corneas, implying prolonged survival of immune effector cells and therefore an extended inflammatory response (Huang and Hazlett, 2003).

Several studies have shown that host response to bacterial and fungal pathogens infecting the cornea appear to converge into common biological pathways as disease progresses (Karthikeyan et al., 2011, 2013). However, there remains a paucity of data on the specific molecular mechanisms within these pathways in human MK. In order to better understand the immunopathogenesis of this disease, we have investigated the human corneal transcriptome in bacterial keratitis (BK) and fungal keratitis (FK) compared with the normal non-infected cadaveric cornea as the control (C), using Illumina HumanHT-12 v4 microarrays. We then validated our findings in a separate cohort of MK patients with an earlier stage of disease using real time quantitative reverse transcriptase PCR (RTqPCR).

## Methods

This study was carried out in accordance with the recommendations of the Ethical Guidelines for Biomedical Research from The Indian Council of Medical Research. The protocol was approved by the Ethics Committees of the London School of Hygiene and Tropical Medicine (ref. no. 6118), Aravind Eye Care System (application no. IRB2011003CLI), and the Indian Council of Medical Research (ref. no. 53/2/oph/indo-foreign/12/NCDII). All subjects and relatives of the deceased (for inclusion of cadaver corneas) gave written informed consent in accordance with the Declaration of Helsinki.

### Participant enrolment: microarray study

Adult subjects (aged ≥ 18 years) undergoing corneal transplantation at Aravind Eye Hospital (AEH) for culture-positive MK were enrolled into the study between January 2012 and 2013. Corneal scrapes were taken from the ulcer at presentation for microbiological culture. Sociodemographic data and ulcer clinical feature data were recorded into a standardized study form on the day of or day before surgery. Immediately after surgical excision, the corneal tissue was cut into four quadrants through the center of the ulcer using sterile technique. Two adjacent quadrants were placed into RNALater (Ambion, TX), stored at 4°C for 24 h, then −80°C until RNA extraction. The remaining two quadrants were formalin-fixed and paraffin wax embedded for an additional experiment. Thirteen adult cadaver corneas that were not eligible for corneal transplantation (due to inadequate endothelial cell count) and with no evidence of pathology on slit lamp examination were collected from Aravind Eye Bank between January 2012 and 2013 and preserved in RNAlater as described above.

### Participant enrolment: validation cohort

Between March 2012 and February 2013, we recruited an independent series of consecutive patients as defined as all patients who presented daily to the cornea clinic at AEH with culture-positive BK or FK and with a moderate/large corneal ulcer (defined as stromal infiltrate ≥ 3 mm in longest diameter and extending >1/3 into the corneal stroma as assessed by slit lamp biomicroscopy) who met the study inclusion/exclusion criteria. Sociodemographic/clinical data were recorded in the standardized study form. After application of a 0.5% proparacaine anesthetic eyedrop (Aurocaine, Aurolab, Madurai, India), a sterile Dacron swab (Puritan Medical, ME) was gently swept across the base and leading edge of the ulcer, then immediately placed into RNAlater. This was kept at 4°C for 24 h, then stored at −80°C until RNA extraction. Then a corneal scrape was taken from the ulcer base and leading edge and material transferred on to two glass slides and two sterile agar plates (blood and potato dextrose agar) for microbiological diagnosis.

### Microbiological diagnosis

For microscopy, the slides stained with gram stain (for bacteria) and 10% potassium hydroxide (to aid in identification of fungal filaments) and examined by an experienced microbiologist. For culture, blood agar plates were incubated at 37°C for 2 days, and potato dextrose agar plates at 27°C for 1 week; agar plates were examined daily for any growth. A positive culture was defined as growth of the same organism on both solid media, or semi-confluent growth at the inoculation site in one solid medium matching the organism identified on microscopy; organisms grown were identified using methods described elsewhere (Wilhelmus et al., 1994). For fungal speciation, we used colony morphology and characteristic microscopic appearances of lactophenol cotton blue-stained hyphae and conidia, as described elsewhere (Thomas, 2003; Guarner and Brandt, 2011). Any culture and microscopy negative corneal ulcers were not included in the study.

### RNA extraction

Total RNA was extracted from corneal tissue using TRIzol (Invitrogen, Carlsbad, CA) after tissue was ground in liquid nitrogen. RNA concentration was estimated by fluorescence (Qubit, Invitrogen). Any microarray samples with <70 ng/μL of RNA underwent speed vacuum concentration (Eppendorf Concentrator Plus, Hamburg, Germany). RNA purity was assessed using Agilent Bioanalyzer RNA 6000 Nanochip (Agilent Technologies Inc., Palo Alto, CA) and samples with RNA integrity number values ≥7 and/or intact 18S and 28S RNA were selected for microarray. For corneal swabs from the validation cohort, total RNA was extracted using the RNeasy micro kit (Qiagen, Netherlands) and RNA quantity estimated using Nanodrop ND-1000 spectrophotometer (ThermoScientific, Waltham, MA).

### Microarray experiment

Total RNA (150 ng) from the 30 samples that passed RNA quality control (QC) measures described above underwent conversion to biotin labeled a-RNA (Target Amp Nano-g Biotin a-RNA labeling kit, EpiCenter Biotechnologies, Madison, WI) and was hybridized on to Illumina HumanHT-12 v4 beadchips (Illumina Inc., San Diego, CA) as per the manufacturer's protocol (Illumina, 2010). Disease and control samples were randomized between the three beadchips used. Fluorescence intensities were imaged with the BeadArray reader (Illumina Inc.). Each beadchip contained 47,231 probes covering the whole-genome and known splice variants. The definition of a probe in the Illumina HT-12 v4 beadchip is a 50-mer sequence-specific oligonucleotide that is designed to recognize a single gene with sequences originating from the National Center for Biotechnology Information Reference Sequence (NCBI) RefSeq Release 38 (November 7, 2009) and legacy UniGene content (Illumina, 2010).

The microarray raw and normalized data are available at NCBI Gene Expression Omnibus repository under accession number GSE58291. QC checks for sample-independent and sample-dependent controls were performed in GenomeStudio version 3.1 (Illumina Inc.) and data exported to Lumi in Bioconductor in R for analysis (Du et al., 2008). Pairwise comparisons were defined as BK or FK vs. controls, and BK vs. FK. Probe-level raw data were divided into subsets according to these pairwise comparisons and only samples involved in a given comparison were included in that analysis. Data were filtered to remove non-expressed probes (probes whose fluorescence was not statistically significantly different to negative control probes, i.e., *p* > 0.01). Variance stabilizing transformation followed by quantile normalization were applied to reduce variation due to non-biological differences (Lin et al., 2008b). Prior to normalization, samples were ordered by strength of their pairwise correlations using agglomerative hierarchical clustering with complete linkage to identify outliers, and also principal component analysis was used with data from all samples to again identify outlying samples. Fold change (FC) was calculated as the antilog_2_ of the difference between the mean normalized expression intensity for each gene in each group in the pairwise comparison.

### RTqPCR experiment

RTqPCR was used to validate the microarray findings using RNA from a) the same microarray samples (except 1 sample with inadequate RNA remaining, M20 in FK) and b) samples from the validation cohort. For the RTqPCR, 48 genes were chosen from the most differentially expressed genes in the microarray using a literature search method to identify genes associated with the host response to bacterial/fungal infection or MK, and also included three genes (*IFNG, IL12B, IL17A*) that were not among the microarray differentially expressed genes (DEGs) but were considered to be of potential biological importance in MK. Three housekeeping genes (*GAPDH, HPRT1, RPP30*) were also included. Primers were selected from Taqman (Life Technologies, New York, USA) gene expression assays (Table S1) and pre-loaded into custom Taqman Low Density Array (TLDA) cards (Life Technologies) with eight samples per card. Technical replicates were run for eight randomly selected samples from the validation cohort and all microarray samples. Total RNA (100 ng) from each sample was converted to cDNA (Takara PrimeScript 1st strand cDNA synthesis kit, Takara Bio, Otsu, Shiga, Japan) in a 20 μL reaction volume; for samples with total RNA <100 ng (*n* = 25), all sample RNA was used. TaqMan (Life Technologies) gene expression mastermix (50 μL), and cDNA were made up to 100 μL with RNAse-free water and loaded into TLDA cards, with disease and control samples randomized to be present on each card. RTqPCR was performed in the ABI 7900HT PCR thermal cycler (Life Technologies). Thermal cycling conditions were 50°C for 2 min; 94.5°C for 10 min (denaturation); 40 cycles of 97°C for 30 s then 59.7°C for 1 min (for annealing and extension). Raw cycle threshold (C_T_) data were collected in SDS RQ manager (Life Technologies) with baseline automatically detected. For samples run in duplicate, the arithmetic mean of C_T_ values was used in data analysis. C_T_ values were normalized to GAPDH as this housekeeping gene showed the best expression profile for all samples. Samples were considered to have failed gene expression if GAPDH was not expressed (i.e., C_*T*_ = 40) and median C_T_ for all 48 genes was > 37, and were excluded from downstream analysis. FC was calculated as the mean normalized expression between groups, using 2^−(ΔCT(meangroup1)−ΔCT(meangroup2))^.

### Statistical analysis

Wilcoxon rank-sum test was used to compare clinical data in each group in Stata v12.1 (StataCorp, Texas, USA). Microarray and RTqPCR pairwise comparisons were assessed for statistical significance using empirical Bayes moderated *t*-tests (Limma package in Bioconductor in R) (Smyth, 2004). *P*-values were adjusted for multiple comparisons using the Benjamini-Hochberg false-discovery rate (FDR) (Benjamini and Hochberg, 1995).

### Differential expression analysis

Differentially expressed genes were defined as FDR-adjusted *p*-value < 0.05 and FC ≥ 4 for upregulation or FC < −4 for downregulation. For genes with multiple probes, the probe with the greatest absolute value of the FC was selected to represent the gene for all downstream analysis. FCs from microarray and RTqPCR were evaluated with Spearman's rank correlation since data were not normally distributed, as confirmed by the Shapiro-Wilk test (Stata v12.1, StataCorp). Genes that were non-expressed in the microarray were not included in the correlation calculations: *IL17A* and *IL12B* for all comparisons, and *IFNG* for FK vs. C. The list of differentially expressed genes was analyzed for gene ontology (GO) terms using Database for Annotation, Visualization, and Integrated Discovery (DAVID) v6.8 (Huang et al., 2009).

### Protein-protein interaction (PPI) network analysis

A human PPI network was constructed using the HIPPIE v2.0 database (Schaefer et al., 2012) and visualized with Cytoscape version 3.4.0 (http://www.cytoscape.org) for differentially expressed genes unique to BK or FK as well as those genes common to both infections. The Molecular Complex Detection (MCODE) plugin was used within Cytoscape to assess highly interconnected clusters of proteins using default parameters (Degree Cutoff: 2, Node Score Cutoff: 0.2, K-Core: 2) (Bader and Hogue, 2003). Degree was defined as the number of connections that each gene had, and degree cutoff was defined the minimum number of connections allowed for each gene to be included in the network (Bader and Hogue, 2003).

### Network analysis: Miru

Normalized expression intensities for each gene expressed in the BK, FK, and C samples in the microarray experiment (*n* = 24,418 probes) were used to generate a Pearson correlation matrix in Miru (Theocharidis et al., 2009). As described above, for genes with multiple probes, the probe with the greatest absolute value of the FC was selected to represent the gene for the network analysis. The three samples found to be outliers in hierarchical clustering were excluded. A network graph was created with each gene drawn as a “node.” The line drawn between two highly correlated nodes (i.e., correlation coefficient ≥ 0.9) is known as an “edge.” The Markov Cluster Algorithm (MCL) was used to highlight natural clusters of the most highly correlated nodes, using standard settings in Miru and smallest cluster size of four nodes (Enright et al., 2002). Gene lists from each cluster were explored for GO term enrichment using DAVID v6.8.

## Results

### Socio-demographic features and microbiology

For the microarray study, a total of 47 participants were enrolled (*n* = 19 controls, *n* = 10 BK, *n* = 18 FK). Corneal tissue from 16 of these participants failed initial RNA QC measures (*n* = 6 controls; *n* = 2 BK: 1 *S. pneumoniae* and 1 *P. aeruginosa*; *n* = 9 FK: 8 *Aspergillus* sp. and 1 *Fusarium* sp). Corneal tissue from the remaining 30 participants passed QC and underwent microarray analysis (*n* = 13 C, *n* = 9 FK, *n* = 8 BK). Hierarchical clustering revealed three outlier specimens that were excluded from further analyses (*n* = 1 BK, *n* = 1 FK, *n* = 1 C). For the validation cohort with earlier stage of disease, 205 participants were enrolled (*n* = 185 FK and 20 BK); 22 of these samples failed RTqPCR QC (*n* = 20 FK, *n* = 2 BK) and were excluded.

Table 1 summarizes socio-demographic and clinical features of the final 183 validation cohort and 27 microarray cohort participants. The control group had an older median age (*p* ≤ 0.001) compared to the other microarray participants, since most cadaver corneas were from deceased older subjects. In the microarray cohort, the symptom duration, ulcer size and visual acuity at presentation was similar in both bacterial and fungal ulcers (Table 1; full details Table S2). All bacterial ulcers in the microarray cohort had corneal perforation, compared to only three in the fungal group; this reflects the different indications for performing corneal transplantation in these two groups. The validation cohort had less severe disease compared with participants in the microarray study, as evidenced by significantly better visual acuity and smaller ulcer size at presentation as well as fewer corneal perforations (Table 1).

**Table 1:** **Summary of clinical and sociodemographic data of study participants**.

	**Controls (*n* = 12)**	**Bacterial keratitis (BK)**			**Fungal keratitis (FK)**			**BK vs. FK**	
		**Microarray Cohort (*n* = 7)**	**Validation Cohort (*n* = 18)**	***p*-value**	**Microarray Cohort (*n* = 8)**	**Validation Cohort (*n* = 165)**	***p*-value**	**Microarray cohort *p*-value**	**Validation Cohort *p*-value**
Gender, No. Male (%)	7 (58%)	7 (100%)	13 (72%)	0.12	4 (50%)	105 (64%)	0.44	0.03	0.47
Age, median (IQR), years	82 (76.5–90)	67 (60–75)	55 (35–62)	0.02	52.5 (43–62.5)	50 (36.5–58)	0.44	0.04	0.41
Symptom duration, median (range), days	–	15 (7–20)	10 (2–90)	0.19	14.5 (2–43)	7 (1–90)	0.12	1.00	0.12
Uncorrected visual acuity^*^, median (IQR)	–	1.9 (1.8–1.9)	1.7 (1.7–1.8)	0.01	1.85 (1.8–1.9)	1.8 (0.56–1.8)	<0.01	0.62	0.86
Median stromal infiltrate size^**^ (mm), (IQR)	–	7.0 (5.7–8.5)	3.8 (3.2–5.1)	<0.01	5.8 (4.3–9.2)	4.4 (3.5—5.5)	0.04	0.68	0.58
Perforation	–	7 (100%)	1 (5%)	0.01	3 (38%)	19 (11%)	<0.01	0.01	0.44

* *Visual Acuity measured in logMAR units:>1.0, unable to read any letters on chart; 1.8, able to count fingers only; 1.9, able to see hand movements only; 2.0, perception of light only*.

** *Stromal infiltrate size calculated as geometric mean of longest stromal infiltrate diameter and its perpendicular measurement (mm)*.

*All p-values generated by comparing microarray study participants vs. validation cohort, within bacterial or fungal keratitis groups*.

For the microarray study, bacterial ulcers were positive for *S. pneumoniae* (*n* = 6) and *P. aeruginosa* (*n* = 1), and fungal ulcers grew *Fusarium* sp. (*n* = 5), *Aspergillus flavus* (*n* = 1), *Aspergillus terreus* (*n* = 1), and *Lasiodiplodia* sp. (*n* = 1). In the validation cohort, most ulcers were fungal (*n* = 165) rather than bacterial (*n* = 23). The commonest fungi were *Fusarium* sp. (*n* = 69) and *A. flavus* (*n* = 25) and among the bacterial ulcers, the most frequent organism was *S. pneumoniae* (*n* = 8; Table 2).

**Table 2:** **Microbiological results of corneal ulcers**.

	**Microarray participants**	**Validation cohort**
	**(*n* = 15)**	**(*n* = 183)**
**BACTERIA**		
*Streptococcus pneumoniae*	6 (40%)	8 (4.4%)
*Pseudomonas aeruginosa*	1 (6.7%)	3 (1.6%)
*Nocardia* sp.	–	3 (1.6%)
*Streptococcus viridans*	–	2 (1.1%)
*Aeromonas* sp.	–	1 (0.5%)
*Staphylococcus epidermidis*	–	1 (0.5%)
**FUNGI**		
*Fusarium* sp.	5 (33.3%)	69 (37.7%)
*Aspergillus flavus*	1 (6.7%)	25 (13.7%)
*Aspergillus terreus*	1 (6.7%)	2 (1.1%)
*Aspergillus fumigatus*	–	4 (2.2%)
*Lasiodiplodia* sp.	1 (6.7%)	2 (1.1%)
*Curvularia* sp.	–	5 (2.7%)
*Exserohilum* sp.	–	5 (2.7%)
*Alternaria* sp.	–	1 (0.5%)
*Bipolaris* sp.	–	1 (0.5%)
*Cylindrocarpon* sp.	–	1 (0.5%)
Unidentified dematiaceous fungus	–	9 (4.9%)
Unidentified hyaline fungus	–	13 (7.1%)
Microscopy positive (no growth)	–	28 (15.3%)

### Microarray study: global gene expression profile and differential expression analysis

**Figure 1:**
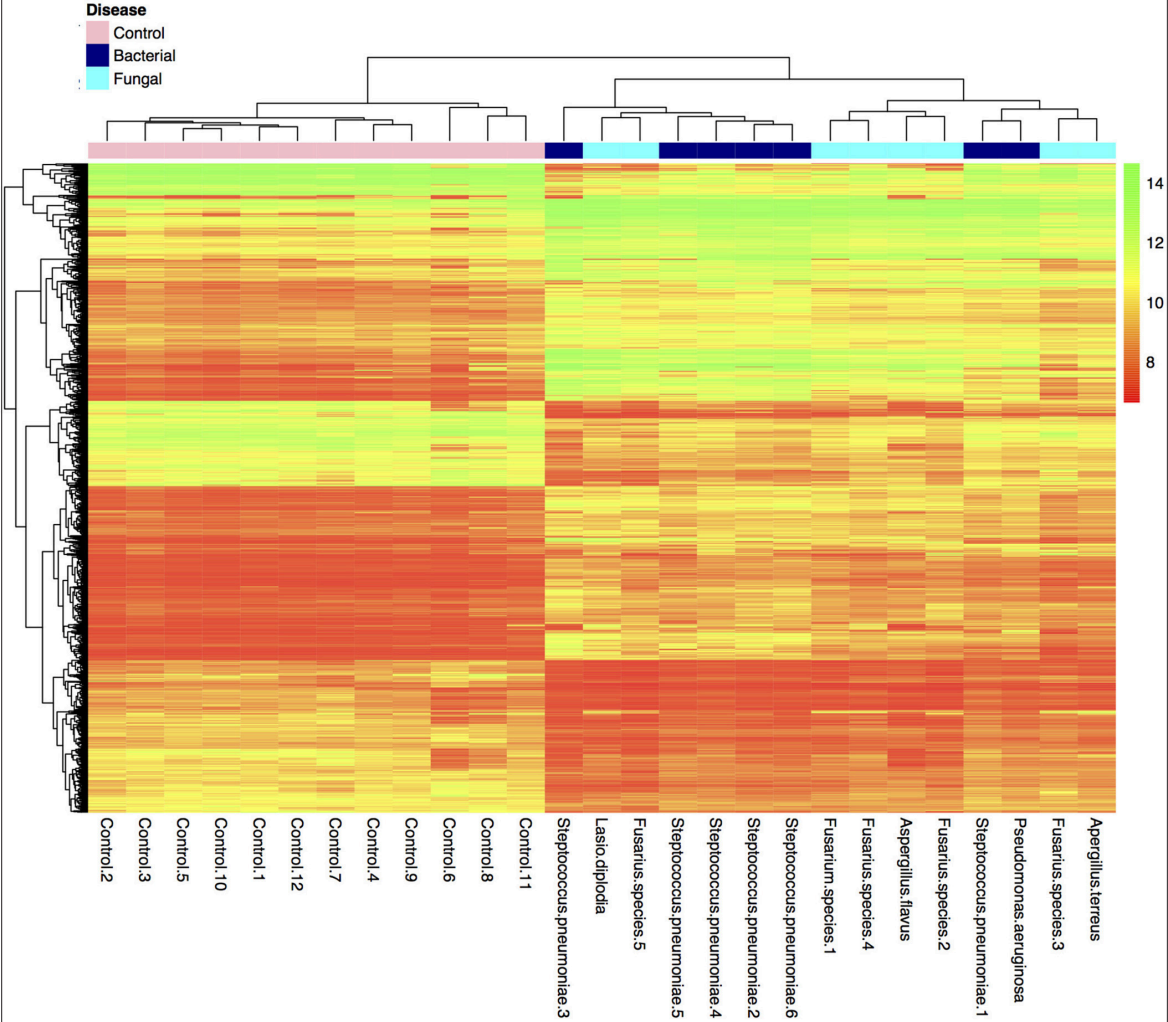
**Plot of hierarchical clustering of log_2_ fold changes of differentially expressed genes from the final bacterial keratitis, fungal keratitis, and control samples used in the microarray experiment (*n* = 27)**. (Color scale red = log_2_FC of 8, green log_2_FC of 14). Bacterial and fungal ulcer samples are interspersed, but normal control tissue samples cluster together in the hierarchical cluster plot of the differentially expressed genes only. The whole-genome level expression profile of raw data for all samples is shown in Figure S1.

Many genes (>17,000) were found to be expressed in the BK and FK vs. C comparisons (Table 3). Hierarchical clustering of transcriptome data revealed a clear difference in infected vs. control samples by their global gene expression profile using differentially expressed genes only (Figure 1), but there was no clear distinction between bacterial and fungal samples. Principal components analysis using whole-genome level expression also shows that control samples cluster together, but bacterial and fungal keratitis samples are interspersed (Figure S1). Differential expression analysis revealed a large number of genes to be differentially expressed in both bacterial and fungal ulcers compared to control tissue (shown in Table S3).

**Table 3:** **Summary of expressed genes in microarray pairwise comparisons (FK, Fungal Keratitis; BK, Bacterial Keratitis; C, Controls)**.

	**FK vs. C**	**BK vs. C**	**BK vs. FK**
No. of expressed genes	17,753	17,434	17,120
*p* < 0.05	6,414	7,825	18
*p* < 0.01	4,119	5,384	0
No. of differentially expressed genes^*^	389	524	0
Up-regulated	291	361	0
Down-regulated	98	163	0

* *Genes were considered differentially expressed if FDR-adjusted p-value < 0.05 and fold change was < –4 or ≥4*.

### Differentially expressed genes common to bacterial and fungal keratitis

Many of the differentially expressed genes were common to both BK and FK (*n* = 339; 250 upregulated and 89 downregulated; Table S4). Using this gene list with DAVID, the most significant GO terms enriched within the biological process category were: the inflammatory response, the immune response, and neutrophil chemotaxis (Table 4), however there is a high degree of overlap of the genes that are present in these three GO terms (i.e., high redundancy). Additional GO terms that were significantly enriched are also shown in Table S4, and include regulation of the immune response, cytokine response, phagocytosis and cell-cell adhesion terms. Within the immune response term, the most highly upregulated cytokine genes included *IL1B* (along with its activator, the inflammasome *NLRP3*), Oncostatin M (*OSM*) and *TNF*, all of which had higher fold changes in bacterial ulcers. Many chemokine genes were upregulated (*CCL2, CCL3, CCL3L1, CCL4L1, CCL5, CCL7, CCL8, CCL13, CCL20*; *CXCL5, CXCL6*) and were found in the significantly enriched chemokine signaling pathway, chemokine activity, or chemokine receptor activity terms. Multiple GO terms associated with leukocyte chemotaxis were enriched and included upregulated genes promoting cell adhesion (e.g., the integrins *ITGB2, ITGAM, ITGAX*) or cell migration via actin filament organization (*HCK, FGR*), or pseudopodia formation (*AQP9*). PRR genes were also upregulated: *CD209 (DCSIGN), TLR2* (and its synergists *NOD2* and *MARCO*), *TLR4* (and co-receptor *CD14*), *TLR8*, as well as *SYK*, a TLR downstream signaling molecule. Elements of the adaptive immune system were also among the differentially expressed genes. The gene coding for the IL7 receptor (*IL7R*) present on naïve and memory T-cells, was strongly upregulated especially in BK. There was also indirect evidence of Th17 activity with high expression of genes coding for the Th17-chemokine *CCL2*. IFN-gamma activity may have been occurring since we observed increased gene expression for IFNG-induced genes (*IFI30, ISG15*). Serglycin, *SRGN*, the main component of cytotoxic T-cell, and NK cell dense granules also had upregulated gene expression.

**Table 4:** **Top three most significantly enriched biological process Gene Ontology (GO) terms for microarray pairwise comparisons**.

	**GO terms**	**Fold enrichment**	***P*-value**
Genes common to both bacterial and fungal keratitis compared to control tissue	GO:0006955: immune response	4.6	5.37 E-50
	GO:0006952: defense response	4.3	2.38 E -42
	GO:0002682: regulation of immune system process	4.4	9.68 E-19
Genes unique to bacterial keratitis	GO:0006954: inflammatory response	6.3	3.63 E-13
	GO: 0002682: regulation of immune system process	5.3	6.89 E-13
	GO:0001816: cytokine production	8.7	1.82 E-12
Genes unique to fungal keratitis	GO:1901700: response to oxygen-containing compound	4.3	2.9 E-06
	GO:0000302: response to reactive oxygen species	11.9	1.2 E-04
	GO:0009605: response to external stimulus	3.0	1.5 E-04

*Analyses performed in DAVID v6.8 (all p-values unadjusted)*.

The defense response term was also found to be a significantly enriched biological processed. Genes that were expressed with high fold changes within this pathway included those in the complement system (*C1QC*; the receptor *C3AR*; complement activators *Ficolin 1, FCN1*, and FC-gamma receptors). However, the complement regulator gene, *Complement Factor H* (*CFH*), was downregulated. Genes involved in multiple microbial killing mechanisms were upregulated including those encoding antimicrobial peptides (*LYZ, DEFB4*), NADPH oxidase subunits (*NCF2, NCF4, CYBB, RAC2*), and phagolysosome components (late-endosome associated *SLC11A1*). Genes promoting ROS detoxification (*SOD2, CD53, IFI30*) and regulating excess ROS production were also upregulated (*EFHD1*).

Enriched cellular component GO terms included the extracellular space, region or exosome, the extracellular matrix (ECM) and the cell surface. Many MMP genes were found within these terms (i.e., *MMP1, MMP7, MMP9, MMP10, MMP12*), especially *MMP9*, which had the highest FC in both BK and FK (91 FC in BK, adj *p* = 3.64 E-12; 64 FC in FK, adj. *p* = 6.10 E-11). With regards to the corneal epithelium, we observed upregulation of genes from the epithelial differentiation complex such as keratins unique to the wound-healing phenotype (*KRT6B, KRT16*), and antimicrobial peptides (*PI3, S100A7, S100A8, S100A9*). Genes promoting stability of the epithelium such as *ASIP*, (associated with epithelial cell adhesion) and some corneal epithelial keratins (*KRT3, KRT12*) were all downregulated. For the corneal stroma, the gene for type 3 collagen, associated with wound healing, was also upregulated (*COL3A1*). However, keratocyte gene markers were downregulated, e.g., the main stromal proteoglycan keratocan (*KERA*), its sulphation enzyme *CHST6*, and the corneal crystallins (*ALDH1A1, ALDH3A1*). The myofibroblast gene marker alpha smooth muscle actin was not a month the differentially expressed genes in BK or FK, and the receptor that promotes myofibroblast differentiation (*TGFBR3*) was downregulated.

### Differentially expressed genes unique to BK

There were 185 uniquely differentially expressed genes in the BK samples (*n* = 111 upregulated, *n* = 74 downregulated). GO analysis identified the three most significant main biological processes to be the inflammatory response, regulation of the immune system, and cytokine production, which again contain many genes that overlap among all three GO terms (Table 4).

The neutrophil chemoattractant *CXCL2* was the most highly upregulated chemokine gene in the BK samples. Other pro-inflammatory genes that had increased expression included cytokines/cytokine receptors (i.e., *IL1A, IL1R2, IL6, IL18RAP*). Also, the gene for the PRR, *TLR4*, was upregulated.

*HIF1A* was one of the most highly upregulated genes in BK that was also a transcription factor. We also detected upregulation of multiple *HIF1A*-induced genes and these were involved in cellular processes such as mitochondrial fusion (*GNG2*).

Further GO analysis of the differentially expressed genes revealed that the main molecular function was receptor binding and cytokine activity. The main cellular component containing most of the differentially expressed genes according to GO analysis was the extracellular region. Examples of genes found within this category were claudin 5 (*CLDN5*) the endothelial cell tight junction, fibulin 2 *FBLN2*, that contributes to corneal elasticity and also a Descemet's membrane (DM) collagen (*COL8A1, COL8A2*); all of these genes were downregulated in the BK samples.

### Differentially expressed genes unique to FK

Fewer genes were differentially expressed uniquely in FK (*n* = 50; 41 upregulated and 9 downregulated). GO analysis highlighted the main biological processes to be response to reactive oxygen species or oxygen-containing compounds (Table 4). The response to external stimulus GO term was also found to be significant in the FK samples, and included selected chemokines (e.g., *CCL22, CXCL10, CXCL13*) that were all upregulated. In addition, other biological process GO terms that were enriched included keratinization and epithelial cell differentiation (Table S4), that contained genes known to promote corneal re-epithelialization such as the cornified envelope proteins (*SPRR2A, SPRR2D, SPRR2F*, and *SPRR3*; Suprabasin, *SBSN*), which were all upregulated. Genes involved in epithelial adhesion such as the tight junction component desmoglein 1 (*DSG1*) were downregulated.

The GO terms enriched in the molecular function category were mainly associated with antioxidant activity, oxidoreductase activity or peroxidase activity, such as glutathione peroxidase 2 (GPX2) and the hemoglobin subunit genes *HBA2* and *HBB* that were upregulated in FK and present in many of these GO terms.

The cellular component GO terms that were enriched for FK were predominantly extracellular and included the cornified envelope, extracellular vesicle/exosome and the extracellular matrix (Table S4). Many genes overlapped between these GO terms, including the corneal collagens (*COL1A1, COL5A1*), a corneal crystallin (*ALDH3A2*), and an integrin (*ITGB7*).

### Microarray probes differentially expressed between bacterial and fungal keratitis

No genes were differentially expressed in the BK vs. FK comparison. The most upregulated gene was *SMPDL3A*, a nucleotide phosphodiesterase present in macrophages, with a fold change of 2.27 (fdr-adjusted *p* = 0.048) in BK samples compared to FK. The most downregulated gene was *ACVRL1*, a receptor in the TGF-beta signaling pathway, that had a fold change of 0.41 in BK vs. FK (fdr-adjusted *p* = 0.014; Table S3).

### Network analysis: MCODE protein-protein interaction and network co-expression

The protein-protein interaction network of all BK DEGs identified the most inter-connected genes as those involved in actin filament reorganization (*BTK, FGR, HCK, LYN, PLCG2, KIT*; see Figure S2). The remaining five clusters detected mainly genes involved in the immune response: chemokines (*CCL22, CCL5, CCR1, CXCR4, CXCL8*), TLR4 signaling (*TLR4, NOD2, IRAK2, IRAK3, RIPK2*), macrophage activity (*CCL7, CCL2, MMP1*), the cytokine *IL1B*, and the final cluster contained the transcription factor *BATF* (promotes Th17 differentiation) and *HLF* (protects against oxidative stress). For FK, MCODE identified only a single cluster which included the same chemokines as detected in BK (*CCL22, CCL5, CCR1, CXCR4, CXCL8*; shown in Figure S2).

Using all of the DEGs for BK and FK, a network co-expression graph was generated in Miru which consisted of 513 nodes and 18,592 edges. The most highly connected genes formed 7 clusters (found using Markov chain clustering algorithm - see Figure S2). Exploration of GO enrichment for each cluster (detailed in Table S5) showed several associated with the immune response, in particular leucocyte migration (cluster 1), cytokine production (cluster 1), or cytokine-mediated signaling pathway involvement (cluster 4) and regulation of T-cell activation (cluster 7). Other GO terms enriched in the remaining clusters included wound healing terms (blood vessel morphogenesis in cluster 2; lymphoid progenitor cell and epithelial cell differentiation in cluster 3; cytoskeleton organization in cluster 5) as well as protein folding associated with cytoplasmic vesicles (cluster 5).

### Validation of microarray results using RTqPCR

**Figure 2:**
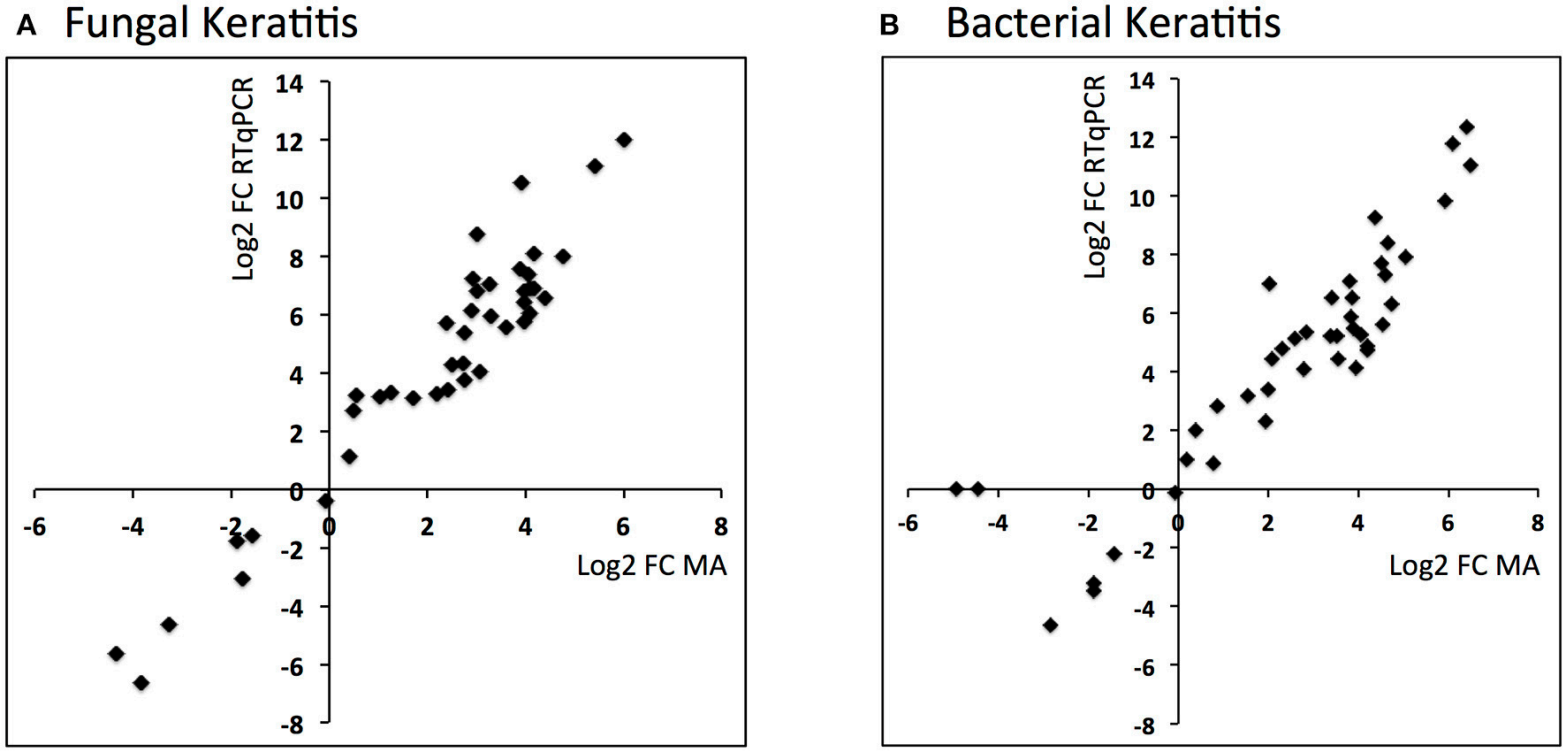
**Scatter plots showing log_2_ fold changes for corneal ulcer tissue analyzed using microarray vs. RTqPCR for (A)** fungal keratitis and **(B)** bacterial keratitis vs. controls (Spearman's rho 0.9, *p* < 0.0001 for both).

Comparison of the RTqPCR results for BK and FK vs. control, and vs. each other, for the microarray cohort and the validation cohorts are shown in Tables 5, 6 respectively. For RNA extracted from corneal tissue used in both the microarray and RTqPCR experiments, we found a high correlation between expression values for the genes tested (Spearman's rho 0.90 for BK and 0.90 for FK samples, *p* < 0.0001 for both; Figure 2). We also found a high correlation for FK (Spearman's rho 0.74, *p* < 0.0001) and moderate correlation for BK (Spearman's rho 0.66, *p* < 0.0001) when comparing the gene expression of the corneal tissue used in the microarray experiment with that of corneal ulcers with late stage disease in the validation cohort of patients (symptom duration > 7 days; *n* = 12 for BK, *n* = 71 for FK). When comparing early stage disease in the validation cohort (symptom duration ≤ 7 days; *n* = 5 for BK, *n* = 94 for FK) with corneal tissue in the microarray samples, we found the top three genes with the highest fold changes were *IL12B, IL23A* and *IL18* in BK, and *IL17A, IL23A* and *IFNG* in FK (Table S6). Within the validation cohort, there were no differentially expressed genes when comparing early vs. late stage disease or BK with FK samples.

**Table 5:** **RTqPCR differential expression analysis results using RNA from microarray study participants**.

**Category**	**Gene**	**BK vs. C**		**FK vs. C**		**BK vs. FK**	
		**FC**	**FDR-adjusted *p*-value**	**FC**	**FDR-adjusted *p*-value**	**FC**	**FDR-adjusted *p*-value**
Immune response	*CXCL5*	5284.76	1.15E-08	1458.86	2.97E-07	3.62	9.32E-01
	*CXCL6*	209.81	3.41E-07	151.09	1.34E-06	1.39	9.32E-01
	*CCL20*	93.32	3.32E-04	41.04	2.43E-03	2.27	9.32E-01
	*CCL3L1*	26.57	3.82E-03	118.69	1.09E-04	0.22	9.32E-01
	*CXCL1*	22.06	1.02E-03	8.86	1.48E-02	2.49	9.32E-01
	*CXCL8*	624.37	1.22E-05	191.25	1.54E-04	3.26	9.32E-01
	*IL1B*	240.14	3.22E-05	118.04	1.59E-04	2.03	9.32E-01
	*TNF*	17.36	4.82E-04	9.80	3.96E-03	1.77	9.32E-01
	*IL17A*	8.20	2.36E-02	1.30	7.82E-01	6.33	9.32E-01
	*IL23A*	7.17	3.88E-02	9.48	1.87E-02	0.76	9.32E-01
	*IFNG*	2.01	4.46E-01	1.18	8.47E-01	1.70	9.32E-01
	*IL18*	0.93	9.05E-01	0.76	6.80E-01	1.23	9.32E-01
	*IL12B*	0.55	4.77E-01	3.20	1.72E-01	0.17	9.32E-01
	*TREM1*	136.48	2.77E-06	130.90	3.07E-06	1.04	9.95E-01
	*CYTL1*	0.00	1.15E-08	0.02	1.54E-05	0.10	4.60E-01
	*AQP9*	904.84	3.88E-08	259.07	1.34E-06	3.49	9.32E-01
PRRs	*CLEC4A*	79.40	8.36E-08	66.52	2.97E-07	1.19	9.32E-01
	*CLEC7A*	1.83	3.36E-01	2.19	2.09E-01	0.84	9.32E-01
	*FCER1G*	49.67	1.66E-06	54.44	1.34E-06	0.91	9.76E-01
	*FCN1*	334.06	2.99E-08	271.71	1.32E-07	1.23	9.32E-01
	*FPR1*	17.83	2.03E-04	13.38	6.31E-04	1.33	9.32E-01
	*SCARA3*	0.09	2.33E-03	0.12	6.26E-03	0.76	9.32E-01
	*TLR2*	9.11	1.64E-03	9.06	1.80E-03	1.00	9.95E-01
	*TLR4*	27.73	9.41E-05	9.84	3.96E-03	2.82	9.32E-01
	*MARCO*	159.96	2.73E-07	163.95	3.95E-07	0.98	9.95E-01
	*NLRP3*	37.29	6.68E-05	19.28	6.17E-04	1.93	9.32E-01
ECM	*COL1A1*	4.91	9.18E-02	16.45	4.84E-03	0.30	9.32E-01
	*COL5A1*	10.64	2.06E-02	19.80	4.36E-03	0.54	9.32E-01
	*KERA*	0.00	2.26E-05	0.01	1.52E-04	0.44	9.32E-01
	*KRT6B*	29.45	3.59E-03	94.62	2.15E-04	0.31	9.32E-01
	*ALDH1A1*	0.04	6.68E-05	0.04	4.97E-05	1.09	9.76E-01
MMPs	*MMP1*	93.57	2.77E-06	110.98	1.70E-06	0.84	9.45E-01
	*MMP10*	21.66	4.82E-04	10.67	4.90E-03	2.03	9.32E-01
	*MMP12*	59.43	1.33E-04	110.09	2.58E-05	0.54	9.32E-01
	*MMP28*	0.22	6.19E-02	0.33	1.68E-01	0.68	9.32E-01
	*MMP7*	41.29	1.78E-04	51.95	9.64E-05	0.79	9.32E-01
	*MMP9*	2117.89	7.16E-10	4151.54	8.34E-11	0.51	9.32E-01
	*ADAM19*	34.91	4.58E-05	70.56	3.18E-06	0.49	9.32E-01
Anti-microbial peptides	*PI3*	3551.44	9.58E-08	2181.29	3.95E-07	1.63	9.32E-01
	*S100A7*	127.46	5.71E-04	427.84	4.97E-05	0.30	9.32E-01
	*S100A9*	37.40	1.20E-04	60.60	2.58E-05	0.62	9.32E-01
	*DEFB4B*	45.05	1.92E-03	84.92	4.65E-04	0.53	9.32E-01
Peptidase inhibitors	*SERPINA1*	38.90	5.38E-06	46.80	2.67E-06	0.83	9.32E-01
	*TIMP1*	3.98	5.34E-02	6.61	9.83E-03	0.60	9.32E-01
	*TIMP3*	0.11	3.66E-03	0.29	8.72E-02	0.39	9.32E-01

*BK, Bacterial Keratitis; FK, Fungal keratitis; C, Controls; PRRs, Pattern Recognition Receptors; ECM, Extra-Cellular Matrix; MMPs, Matrix Metallopeptidases*.

**Table 6:** **RTqPCR differential expression analysis results using samples from the validation cohort of microbial keratitis patients**.

**Category**	**Gene**	**BK vs. C**		**FK vs. C**		**BK vs. FK**	
		**FC**	**FDR-adjusted *p*-value**	**FC**	**FDR-adjusted *p*-value**	**FC**	**FDR-adjusted *p*-value**
**Immune response**	*CXCL5*	2792.89	3.21E-16	1564.99	9.69E-21	1.78	0.947
	*CXCL6*	205.85	7.50E-06	196.07	3.68E-08	1.05	0.957
	*CCL20*	1320.91	3.78E-16	656.98	8.32E-20	2.01	0.947
	*CCL3L1*	147.23	1.95E-07	180.04	2.92E-11	0.82	0.947
	*CXCL1*	109.8	2.93E-11	84.25	8.61E-15	1.3	0.947
	*CXCL8*	1035.87	2.15E-14	1272.22	2.15E-21	0.81	0.947
	*IL1B*	959.19	1.44E-13	1296.59	1.32E-20	0.74	0.947
	*TNF*	65.29	1.98E-06	74.92	1.36E-09	0.87	0.947
	*IL17A*	201.64	1.18E-04	218.43	1.27E-06	0.92	0.957
	*IL23A*	1238.8	3.84E-14	1665.71	2.15E-21	0.74	0.947
	*IFNG*	138.09	8.47E-04	129.84	4.11E-05	1.06	0.957
	*IL18*	27.5	2.84E-05	19.34	2.76E-06	1.42	0.947
	*IL12B*	190.28	3.71E-05	182.04	3.73E-07	1.05	0.957
	*TREM1*	206.05	8.59E-10	423.63	4.60E-17	0.49	0.947
	*CYTL1*	0.05	6.46E-03	0.03	1.32E-04	1.46	0.947
	*AQP9*	1167.59	1.29E-13	1591.01	9.69E-21	0.73	0.947
PRRs	*CLEC4A*	135.1	1.03E-09	311.48	1.20E-17	0.43	0.947
	*CLEC7A*	19.42	1.26E-04	24.77	3.23E-07	0.78	0.947
	*FCER1G*	77.22	4.24E-09	145.47	4.32E-16	0.53	0.947
	*FCN1*	268.45	5.35E-10	465.36	1.40E-16	0.58	0.947
	*FPR1*	161.68	1.22E-10	253.77	2.15E-17	0.64	0.947
	*SCARA3*	1.64	6.16E-01	0.93	9.28E-01	1.76	0.947
	*TLR2*	270.16	1.68E-12	497.91	9.69E-21	0.54	0.947
	*TLR4*	76.09	7.50E-06	106.71	2.72E-09	0.71	0.947
	*MARCO*	398.21	1.38E-11	251.09	9.53E-15	1.59	0.947
	*NLRP3*	35.68	3.72E-05	48.95	3.22E-08	0.73	0.947
ECM	*COL1A1*	6.66	2.91E-02	3.21	9.56E-02	2.07	0.947
	*COL5A1*	61.06	5.24E-05	47.83	2.20E-06	1.28	0.947
	*KERA*	0.03	1.28E-02	0.04	3.57E-03	0.8	0.947
	*KRT6B*	2101.97	6.00E-21	2937.8	3.19E-31	0.72	0.947
	*ALDH1A1*	1.8	5.27E-01	0.74	6.80E-01	2.44	0.947
MMPs	*MMP1*	536.49	1.39E-12	565.54	3.04E-18	0.95	0.957
	*MMP10*	459.9	2.93E-11	405.57	1.17E-15	1.13	0.947
	*MMP12*	2791.89	1.07E-08	717.96	3.14E-09	3.89	0.947
	*MMP28*	12.71	1.70E-02	8.39	1.28E-02	1.52	0.947
	*MMP7*	335.79	7.50E-09	296.06	3.26E-12	1.13	0.947
	*MMP9*	7754.12	5.23E-22	11661.09	8.82E-33	0.66	0.947
	*ADAM19*	156.56	7.64E-08	284.19	2.46E-13	0.55	0.947
Anti-microbial peptides	*PI3*	4787.64	6.43E-23	5666.71	8.82E-33	0.84	0.947
	*S100A7*	7119.6	1.44E-13	3891.02	1.90E-17	1.83	0.947
	*S100A9*	831.43	6.43E-23	921.41	8.82E-33	0.9	0.947
	*DEFB4B*	1421.62	6.51E-14	1208.72	2.57E-19	1.18	0.947
Peptidase inhibitors	*SERPINA1*	134.64	1.29E-10	311.83	2.57E-19	0.43	0.947
	*TIMP1*	3.81	1.23E-02	4.4	5.26E-04	0.87	0.947
	*TIMP3*	1.09	9.26E-01	0.59	4.97E-01	1.84	0.947

*BK, Bacterial Keratitis; FK, Fungal keratitis; C, Controls; PRRs, Pattern Recognition Receptors; ECM, Extra-Cellular Matrix; MMPs, Matrix Metallopeptidases*.

## Discussion

In this study, we examined the main genes and biological processes contributing to pathogenesis in human MK. Even though we studied late stage keratitis up to 15 days post-symptom onset, we found a prominence of innate immune pathways among the DEGs in both BK and FK. In the microarray experiment all of the participants with BK experienced corneal perforation. Their corneal gene expression profile reflected this with differential expression of many genes associated with tissue destruction. *MMP9* had the highest fold change among all DEGs for BK in particular but also FK. The high level of *MMP9* gene expression may be due its production by many cell types including corneal epithelial cells (to aid their migration to close the wound), activated keratocytes, infiltrating macrophages and neutrophils, although the latter appear to be the main source (Matsubara et al., 1991; Wong et al., 2002; Mulholland et al., 2005; McClellan et al., 2006; Lin et al., 2008a). MMP9 in addition to destroying Type IV collagen (the main collagen in DM) is able to cleave and activate pro-IL1B, thereby contributing to inflammation in the cornea (McClellan et al., 2006). Other MMPs also had highly upregulated gene expression in both BK and FK, including MMP10, blockade of which improves corneal epithelial healing in diabetic corneas (Saghizadeh et al., 2013). Pharmacological inhibition of MMPs with tetracyclines has shown some success toward halting corneal perforation in both animal models and human MK, and thus may prove to be a potential therapeutic target (Levy and Katz, 1990; McElvanney, 2003).

Laying down of new collagen to rebuild DM did not appear to occur effectively in BK, since the genes for key collagen subunits in DM (collagen type 8) were downregulated (Matsubara et al., 1991; Hopfer et al., 2005; McClellan et al., 2006; Dyrlund et al., 2012). BK tissue compared to FK had a higher proportion of type III collagen gene expression vs. type I, as well as versican, both of which contribute to a weaker wound that is more likely to perforate as explored previously in other tissues but not the cornea (Venkatesan et al., 2000; Eriksen et al., 2002; Andersson-Sjoland et al., 2015). Other factors contributing to weakness of the posterior-most layer of the cornea in BK were downregulation of gene expression of *claudin 5*, a major component of tight junctions between corneal endothelial cells, and *fibulin 2*, that gives elasticity and strength to DM (Nakasaki et al., 2015).

Evidence of corneal epithelial wound healing was present FK more than BK, with increased expression of genes in the cornified envelope protein category. Many genes that are part of the “epithelial differentiation complex” described in epidermal wound healing were found to be upregulated in both BK and FK. Genes such as *PI3, SLPI, S100A7* that are part of this complex also have antimicrobial activity. The broad-spectrum antimicrobial activity of PI3 and SLPI proteins have led to investigation into their potential as treatments for infectious diseases, and could therefore be explored as adjunct antimicrobials for keratitis (Williams et al., 2006).

Within the stroma, corneal keratocytes take on a characteristic expression profile toward a fibroblastic phenotype during healing (Hassell and Birk, 2010). We also observed this in both BK and FK with downregulation of *KERA, TKT, ALDH3A1*, and *ALDH1A1* genes, and upregulation of wound-healing collagen genes (*COL1A1, COL3A1*). Activated keratocytes differentiate into myofibroblasts upon exposure to growth factors such as TGFB, allowing them to express alpha smooth muscle actin and promote wound closure (Hassell and Birk, 2010). We found downregulation of the *TGFBR3* gene in both BK and FK and we did not detect the gene for the myofibroblast marker alpha-smooth muscle actin among the DEGs in BK or FK. The presence of highly upregulated *IL1B* gene in both BK and FK may have played a role in inhibiting the differentiation of fibroblasts to myofibroblasts (Mia et al., 2014).

Upregulation of specific PRRs genes gives an insight into the balance between pro- and anti-inflammatory pathways triggered in BK and FK. The *TLR4* gene was upregulated in BK, as previously reported in corneal scrapings from human BK (Karthikeyan et al., 2011, 2013). However, activation of TLRs may not always bring about a pro-inflammatory response. Chronic TLR4 activation in bacterial infection with persistent lipopolysaccharide produces anti-inflammatory IL10, and in *Aspergillus* sp. infection results in reduced IL1B and IL6 production (Chai et al., 2009; Gurung et al., 2015). Further research is required into this tolerance effect and anti-inflammatory activity of TLRs that may contribute to persistence of pathogen in late stage keratitis.

Although innate immune response genes dominated the BK and FK transcriptome, there were also some DEGs that represented adaptive immune pathways, albeit with lower levels of differential expression. The T-cell associated IL7 receptor gene (*IL7R*) was more highly upregulated in BK samples than in FK. We did not directly detect differential expression of *IL17A-E*, in both BK and FK in the microarray cohort, but we did observe a much higher lever of *IL17A* gene expression in early stage FK samples compared to end-stage FK tissue in the microarray cohort, implying a role for this cytokine in the host response early on in fungal keratitis. Also, in BK, the early stage ulcers had higher expression of the *IL23A* and *IL12B* genes, which together encode the components of the IL23 cytokine, that can maintain Th17 cells. In addition, the *IFNG* gene was also expressed more highly in early stage FK rather than late stage FK tissue. Karthikeyan et al. also found the *IFNG* gene to be differentially expressed in both early and late stage ulcers in human FK, and correlated this to the presence of CD3+ and CD4+ cells in the immunohistochemical analysis of late stage ulcers, implying a Th1 response (Karthikeyan et al., 2011). Further studies are required to more fully explore the balance of Th1 and Th17 responses in early vs. late stages of human MK.

The gene expression profile of BK compared to control tissue showed a much greater degree of differential expression than in FK, with higher fold changes in pro-inflammatory genes. *CXCL2* gene had the highest fold change in BK among the chemokines and this gene promotes persistent neutrophil influx even in later stages of keratitis (Kernacki et al., 2001).

The most prominent gene signature in the BK tissue was the upregulation of the transcription factor *HIF1A* and many of its induced genes. The presence of *HIF1A* appears to be essential for successful resolution of BK in Pseudomonal infection, as siRNA or pharmacological blockade results in less NO production, ineffective bacterial killing and ultimately corneal perforation in a murine model of disease (Berger et al., 2013). It acts as a transcription factor to increase the expression of multiple genes required for the clearance of infection, and as such has been described as a master regulator of innate immunity; *HIF1A* has a preferential effect on promoting activity of neutrophils and macrophages, boosting their antimicrobial activities through sustained glycolysis in hypoxic conditions. This occurs mainly through upregulation of mitochondrial genes, which we also detected in our BK samples. However, *HIF1A* has the opposite effect on T-cells and so may suppress the adaptive immune response (Bhandari and Nizet, 2014). Augmentation of the activity of *HIF1A* or molecules within its pathway early on in the disease process have been explored as a therapeutic option for some infections, but is yet to be investigated in MK (Bhandari and Nizet, 2014).

Both types of network analyses used in this study (PPI and network co-expression analyses) complemented the findings of the differential expression analyses, with the most highly interconnected gene clusters being enriched for immune response GO terms, including aspects of the innate immune response (such as macrophage activity in BK, or TLR4 activity and chemokine production in both BK and FK) as well as the adaptive system (such as transcription factor for Th17 cell differentiation in BK, and regulation of T-cell activation in both BK and FK in the network co-expression analyses). Multiple genes associated with actin filament reorganization were found to be highly interconnected in BK; this may be due to the fact that dynamic actin filament changes are required for many cellular antibacterial functions, such as cell migration and phagocytosis (both of which were in the list of enriched GO terms for DEGs common to BK and FK).

In this study, we used control tissue from donors who were significantly older than the keratitis patients, as tissue from younger donors was prioritized for use in corneal transplantation. Although this may have resulted in reduced cell density, particularly keratocytes and endothelial cells, due to the effect of age (Patel et al., 2001; Sanchis-Gimeno et al., 2005), we believe that the marked differences in gene expression observed between cases and controls are more likely to be driven by the presence of infection, rather than a moderate difference in age. Another limitation of this study is that we have focused on the host but not the pathogen transcriptome. Keratitis-causing pathogens can also contribute to disease pathogenesis through evasion of host immune mechanisms, promotion of host cell apoptosis and production of ECM-destroying enzymes (Burns et al., 1990; Gopinathan et al., 2001). Further studies are needed to more fully explore the host-pathogen interaction in MK. We found a great deal of similarity in the transcriptome between bacterial and fungal samples as demonstrated in the global gene expression profiles. Previous studies have also found an overlap in human gene expression responses from late stage *Aspergillus* and *Fusarium* keratitis as well as *S. pneumoniae* vs. *P. aeruginosa* keratitis (Karthikeyan et al., 2011, 2013). Future studies with a larger sample size may be able to more fully elucidate the time point at which the transcriptomic response to these infections in the cornea begin to converge.

In summary, we have reported the human transcriptional response of late stage corneal ulcer tissue following bacterial and fungal infection in the human cornea, and have focused on many genes that have not been previously explored in keratitis. Our findings provide an initial foundation for further exploration of some of these genes as potential prognostic biomarkers or therapeutic targets to treat this blinding eye disease.

## Author contributions

Conception or design of the work: JC, PS, PL, MH, and MB. Acquisition of data: JC, PS, MS, PL, SE, and NP. Analysis or interpretation of the data: JC, SK, JB, MH, and MB. Manuscript drafting or critical revision: JC, SK, JB, NP, MB, and MH. Final approval of published version: all authors. All authors agree to be accountable for all aspects of the work and will ensure that questions related to accuracy or integrity of any part of the work are appropriately investigated and resolved.

## Funding

This work was funded by the Wellcome Trust (grant no. 097437/Z/11/Z to JC).

### Conflict of interest statement

The authors declare that the research was conducted in the absence of any commercial or financial relationships that could be construed as a potential conflict of interest.

## Supplementary material

Figure S1 **Hierarchical Clustering dendrogram (A)** and Principal Components Analysis plot **(B)** for the raw data for all samples (i.e., all expressed probes prior to normalization) which clearly show that M80, M50, and M73 (shown with an asterix in the PCA plot) cluster away from all other samples in these plots.

Figure S2 **Network maps of the Protein-Protein Interaction Analysis for Bacterial Keratitis vs. Control and Fungal Keratitis vs. Control, as well as the network co-expression analysis for all differentially expressed genes in bacterial and fungal keratitis**.

Table S1 **Details of primers used for RTqPCR TLDA Experiment (Life Technologies, USA)**.

Table S2 **Demographic, Clinical, and Microbiological Data for All Microarray Participants**.

Table S3 **List of differentially expressed genes, fold changes, and false-discovery rate (FDR) adjusted *p*-values in the three microarray pairwise comparisons (FK vs. C, BK vs. C, and BK vs. FK)**.

Table S4 **Gene ontology enrichment analysis for the differentially expressed genes common to both bacterial and fungal keratitis: biological process (BP_FAT), molecular function (MF_FAT), and cellular component (CC_FAT) terms from DAVID v6.8**.

Table S5 **Gene ontology term enrichment analysis for each of the seven most highly connected gene clusters in the network co-expression analysis (using DAVID v6.8)**. Normalized expression intensities of all differentially expressed genes in the bacterial and fungal keratitis vs. control comparisons used to create network graph in Miru).

Table S6 **Comparison of early stage samples from the validation cohort (symptom duration ≤7 days) with microarray tissue samples**.

## References

[B1] Andersson-Sjoland A. Hallgren O. Rolandsson S. Weitoft M. Tykesson E. Larsson-Callerfelt A. K.. (2015). Versican in inflammation and tissue remodeling: the impact on lung disorders. Glycobiology 25, 243–251. 10.1093/glycob/cwu120 25371494 PMC4310351

[B2] Bader G. D. Hogue C. W. (2003). An automated method for finding molecular complexes in large protein interaction networks. BMC Bioinformatics 4:2. 10.1186/1471-2105-4-2 12525261 PMC149346

[B3] Benjamini Y. Hochberg Y. (1995). Controlling the false discovery rate: a practical and powerful approach to multiple testing. J. R. Stat. Soc. Ser. B 57, 289–300.

[B4] Berger E. A. McClellan S. A. Vistisen K. S. Hazlett L. D. (2013). HIF-1alpha is essential for effective PMN bacterial killing, antimicrobial peptide production and apoptosis in *Pseudomonas aeruginosa* keratitis. PLoS Pathog. 9:e1003457. 10.1371/journal.ppat.1003457 23874197 PMC3715414

[B5] Bhandari T. Nizet V. (2014). Hypoxia-Inducible Factor (HIF) as a pharmacological target for prevention and treatment of infectious diseases. Infect. Dis. Ther. 3, 159–174. 10.1007/s40121-014-0030-1 25134687 PMC4269623

[B6] Burns F. R. Paterson C. A. Gray R. D. Wells J. T. (1990). Inhibition of *Pseudomonas aeruginosa* elastase and *Pseudomonas keratitis* using a thiol-based peptide. Antimicrob. Agents Chemother. 34, 2065–2069. 2127341 10.1128/aac.34.11.2065 PMC172000

[B7] Burton M. J. Pithuwa J. Okello E. Afwamba I. Onyango J. J. Oates F.. (2011). Microbial keratitis in East Africa: why are the outcomes so poor? Ophthalmic Epidemiol. 18, 158–163. 10.3109/09286586.2011.595041 21780874 PMC3670402

[B8] Chai L. Y. Kullberg B. J. Vonk A. G. Warris A. Cambi A. Latge J. P.. (2009). Modulation of Toll-like receptor 2 (TLR2) and TLR4 responses by *Aspergillus fumigatus*. Infect. Immun. 77, 2184–2192. 10.1128/IAI.01455-08 19204090 PMC2681752

[B9] Du P. Kibbe W. A. Lin S. M. (2008). lumi: a pipeline for processing Illumina microarray. Bioinformatics 24, 1547–1548. 10.1093/bioinformatics/btn224 18467348

[B10] Dyrlund T. F. Poulsen E. T. Scavenius C. Nikolajsen C. L. Thogersen I. B. Vorum H.. (2012). Human cornea proteome: identification and quantitation of the proteins of the three main layers including epithelium, stroma, and endothelium. J. Proteome Res. 11, 4231–4239. 10.1021/pr300358k 22698189 PMC3411198

[B11] Enright A. J. Van Dongen S. Ouzounis C. A. (2002). An efficient algorithm for large-scale detection of protein families. Nucleic Acids Res. 30, 1575–1584. 10.1093/nar/30.7.1575 11917018 PMC101833

[B12] Eriksen H. A. Pajala A. Leppilahti J. Risteli J. (2002). Increased content of type III collagen at the rupture site of human Achilles tendon. J. Orthop. Res. 20, 1352–1357. 10.1016/S0736-0266(02)00064-5 12472252

[B13] Galarreta D. J. Tuft S. J. Ramsay A. Dart J. K. (2007). Fungal keratitis in London: microbiological and clinical evaluation. Cornea 26, 1082–1086. 10.1097/ICO.0b013e318142bff3 17893539

[B14] Gopinathan U. Ramakrishna T. Willcox M. Rao C. M. Balasubramanian D. Kulkarni A.. (2001). Enzymatic, clinical and histologic evaluation of corneal tissues in experimental fungal keratitis in rabbits. Exp. Eye Res. 72, 433–442. 10.1006/exer.2000.0971 11273671

[B15] Gorscak J. J. Ayres B. D. Bhagat N. Hammersmith K. M. Rapuano C. J. Cohen E. J.. (2007). An outbreak of *Fusarium keratitis* associated with contact lens use in the northeastern United States. Cornea 26, 1187–1194. 10.1097/ICO.0b013e318142b932 18043174

[B16] Guarner J. Brandt M. E. (2011). Histopathologic diagnosis of fungal infections in the 21st century. Clin. Microbiol. Rev. 24, 247–280. 10.1128/CMR.00053-10 21482725 PMC3122495

[B17] Gurung P. Li B. Subbarao Malireddi R. K. Lamkanfi M. Geiger T. L. Kanneganti T. D. (2015). Chronic TLR stimulation controls NLRP3 inflammasome activation through IL-10 mediated regulation of NLRP3 expression and Caspase-8 activation. Sci. Rep. 5:14488. 10.1038/srep14488 26412089 PMC4585974

[B18] Hassell J. R. Birk D. E. (2010). The molecular basis of corneal transparency. Exp. Eye Res. 91, 326–335. 10.1016/j.exer.2010.06.021. 20599432 PMC3726544

[B19] Hopfer U. Fukai N. Hopfer H. Wolf G. Joyce N. Li E.. (2005). Targeted disruption of Col8a1 and Col8a2 genes in mice leads to anterior segment abnormalities in the eye. FASEB J. 19, 1232–1244. 10.1096/fj.04-3019com 16051690

[B20] Huang D. W. Sherman B. T. Lempicki R. A. (2009). Bioinformatics enrichment tools: paths toward the comprehensive functional analysis of large gene lists. Nucleic Acids Res. 37, 1–13. 10.1093/nar/gkn923 19033363 PMC2615629

[B21] Huang X. Hazlett L. D. (2003). Analysis of *Pseudomonas aeruginosa* corneal infection using an oligonucleotide microarray. Invest. Ophthalmol. Vis. Sci. 44, 3409–3416. 10.1167/iovs.03-0162 12882789

[B22] Illumina I. (2010). Whole-Genome Gene Expression Direct Hybridization Assay Guide.

[B23] Karthikeyan R. S. Leal S. M. Jr. Prajna N. V. Dharmalingam K. Geiser D. M. Pearlman E. (2011). Expression of innate and adaptive immune mediators in human corneal tissue infected with Aspergillus or fusarium. J. Infect. Dis. 204, 942–950. 10.1093/infdis/jir426 21828275 PMC3156922

[B24] Karthikeyan R. S. Priya J. L. Leal S. M. Jr. Toska J. Rietsch A. Prajna V.. (2013). Host response and bacterial virulence factor expression in Pseudomonas aeruginosa and *Streptococcus pneumoniae* corneal ulcers. PLoS ONE 8:e64867. 10.1371/journal.pone.0064867 23750216 PMC3672173

[B25] Kernacki K. A. Barrett R. P. McClellan S. Hazlett L. D. (2001). MIP-1alpha regulates CD4+ T cell chemotaxis and indirectly enhances PMN persistence in *Pseudomonas aeruginosa* corneal infection. J. Leukoc. Biol. 70, 911–919. 11739554

[B26] Leal S. M. Jr. Cowden S. Hsia Y. C. Ghannoum M. A. Momany M. Pearlman E. (2010). Distinct roles for Dectin-1 and TLR4 in the pathogenesis of *Aspergillus fumigatus* keratitis. PLoS Pathog. 6:e1000976. 10.1371/journal.ppat.1000976 20617171 PMC2895653

[B27] Levy J. H. Katz H. R. (1990). Effect of systemic tetracycline on progression of *Pseudomonas aeruginosa* keratitis in the rabbit. Ann. Ophthalmol. 22, 179–183. 2114813

[B28] Lin M. Jackson P. Tester A. M. Diaconu E. Overall C. M. Blalock J. E.. (2008a). Matrix metalloproteinase-8 facilitates neutrophil migration through the corneal stromal matrix by collagen degradation and production of the chemotactic peptide Pro-Gly-Pro. Am. J. Pathol. 173, 144–153. 10.2353/ajpath.2008.080081 18556780 PMC2438292

[B29] Lin S. M. Du P. Huber W. Kibbe W. A. (2008b). Model-based variance-stabilizing transformation for Illumina microarray data. Nucleic Acids Res. 36, e11. 10.1093/nar/gkm1075 18178591 PMC2241869

[B30] Matsubara M. Zieske J. D. Fini M. E. (1991). Mechanism of basement membrane dissolution preceding corneal ulceration. Invest. Ophthalmol. Vis. Sci. 32, 3221–3237. 1660857

[B31] McClellan S. A. Huang X. Barrett R. P. Lighvani S. Zhang Y. Richiert D.. (2006). Matrix metalloproteinase-9 amplifies the immune response to *Pseudomonas aeruginosa* corneal infection. Invest. Ophthalmol. Vis. Sci. 47, 256–264. 10.1167/iovs.05-1050 16384971

[B32] McElvanney A. M. (2003). Doxycycline in the management of pseudomonas corneal melting: two case reports and a review of the literature. Eye Contact Lens 29, 258–261. 10.1097/01.icl.0000086490.38331.58 14555906

[B33] Mia M. M. Boersema M. Bank R. A. (2014). Interleukin-1beta attenuates myofibroblast formation and extracellular matrix production in dermal and lung fibroblasts exposed to transforming growth factor-beta1. PLoS ONE 9:e91559. 10.1371/journal.pone.0091559 24622053 PMC3951452

[B34] Mulholland B. Tuft S. J. Khaw P. T. (2005). Matrix metalloproteinase distribution during early corneal wound healing. Eye 19, 584–588. 10.1038/sj.eye.6701557 15332107

[B35] Nakasaki M. Hwang Y. Xie Y. Kataria S. Gund R. Hajam E. Y. (2015). The matrix protein Fibulin-5 is at the interface of tissue stiffness and inflammation in fibrosis. Nat. Commun. 6:8574 10.1038/ncomms9574 26469761 PMC4634219

[B36] Patel S. McLaren J. Hodge D. Bourne W. (2001). Normal human keratocyte density and corneal thickness measurement by using confocal microscopy *in vivo*. Invest. Ophthalmol. Vis. Sci. 42, 333–339. 11157863

[B37] Poole T. R. G. (2002). Aetiology of microbial keratitis in northern Tanzania. Br. J. Ophthalmol. 86, 941–942. 10.1136/bjo.86.8.941 12140229 PMC1771226

[B38] Saghizadeh M. Epifantseva I. Hemmati D. M. Ghiam C. A. Brunken W. J. Ljubimov A. V. (2013). Enhanced wound healing, kinase and stem cell marker expression in diabetic organ-cultured human corneas upon MMP-10 and cathepsin F gene silencing. Invest. Ophthalmol. Vis. Sci. 54, 8172–8180. 10.1167/iovs.13-13233 24255036 PMC3867183

[B39] Sanchis-Gimeno J. A. Lleo-Perez A. Alonso L. Rahhal M. S. Martinez Soriano F. (2005). Corneal endothelial cell density decreases with age in emmetropic eyes. Histol. Histopathol. 20, 423–427. 10.14670/HH-20.423 15736046

[B40] Schaefer M. H. Fontaine J. F. Vinayagam A. Porras P. Wanker E. E. Andrade-Navarro M. A. (2012). HIPPIE: integrating protein interaction networks with experiment based quality scores. PLoS ONE 7:e31826. 10.1371/journal.pone.0031826 22348130 PMC3279424

[B41] Smyth G. K. (2004). Linear models and empirical bayes methods for assessing differential expression in microarray experiments. Stat. Appl. Genet. Mol. Biol. 3, 1–26. 10.2202/1544-6115.1027 16646809

[B42] Srinivasan M. Gonzales C. A. George C. Cevallos V. Mascarenhas J. M. Asokan B.. (1997). Epidemiology and aetiological diagnosis of corneal ulceration in Madurai, south India. Br. J. Ophthalmol. 81, 965–971. 9505820 10.1136/bjo.81.11.965 PMC1722056

[B43] Steuhl K. P. Doring G. Henni A. Thiel H. J. Botzenhart K. (1987). Relevance of host-derived and bacterial factors in *Pseudomonas aeruginosa* corneal infections. Invest. Ophthalmol. Vis. Sci. 28, 1559–1568. 3305411

[B44] Sun Y. Karmakar M. Roy S. Ramadan R. T. Williams S. R. Howell S.. (2010). TLR4 and TLR5 on corneal macrophages regulate Pseudomonas aeruginosa keratitis by signaling through MyD88-dependent and -independent pathways. J. Immunol. 185, 4272–4283. 10.4049/jimmunol.1000874 20826748 PMC3392180

[B45] Theocharidis A. van Dongen S. Enright A. J. Freeman T. C. (2009). Network visualization and analysis of gene expression data using BioLayout Express(3D). Nat. Protoc. 4, 1535–1550. 10.1038/nprot.2009.177 19798086

[B46] Thomas P. A. (2003). Current perspectives on ophthalmic mycoses. Clin. Microbiol. Rev. 16, 730–797. 10.1128/CMR.16.4.730-797.2003 14557297 PMC207127

[B47] Tuft S. J. Matheson M. (2000). *In vitro* antibiotic resistance in bacterial keratitis in London. Br. J. Ophthalmol. 84, 687–691. 10.1136/bjo.84.7.687 10873974 PMC1723533

[B48] Venkatesan N. Ebihara T. Roughley P. J. Ludwig M. S. (2000). Alterations in large and small proteoglycans in bleomycin-induced pulmonary fibrosis in rats. Am. J. Respir. Crit. Care Med. 161, 2066–2073. 10.1164/ajrccm.161.6.9909098 10852789

[B49] Whitcher J. P. Srinivasan M. Upadhyay M. P. (2001). Corneal blindness: a global perspective. Bull. World Health Organ. 79, 214–221. 10.1590/S0042-96862001000300009 11285665 PMC2566379

[B50] Wilhelmus K. R. Liesegang T. J. Osato M. Jones D. B. (1994). Cumitech 13A: Laboratory Diagnosis of Ocular Infections. American Society of Microbiology.

[B51] Williams S. E. Brown T. I. Roghanian A. Sallenave J. M. (2006). SLPI and elafin: one glove, many fingers. Clin. Sci. 110, 21–35. 10.1042/CS20050115 16336202

[B52] Wong T. T. Sethi C. Daniels J. T. Limb G. A. Murphy G. Khaw P. T. (2002). Matrix metalloproteinases in disease and repair processes in the anterior segment. Surv. Ophthalmol. 47, 239–256. 10.1016/S0039-6257(02)00287-4 12052410

