# Supplementary figures and images for: Persistence of Innate Immune Pathways in Late Stage Human Bacterial and Fungal Keratitis: Results from a Comparative Transcriptome Analysis
Source: Front Cell Infect Microbiol. 2017 May 18;7:193. doi: 10.3389/fcimb.2017.00193 (PMC5435826; doi:10.3389/fcimb.2017.00193)

Sample relations based on 47319 genes with sd/mean > 0.1

A)

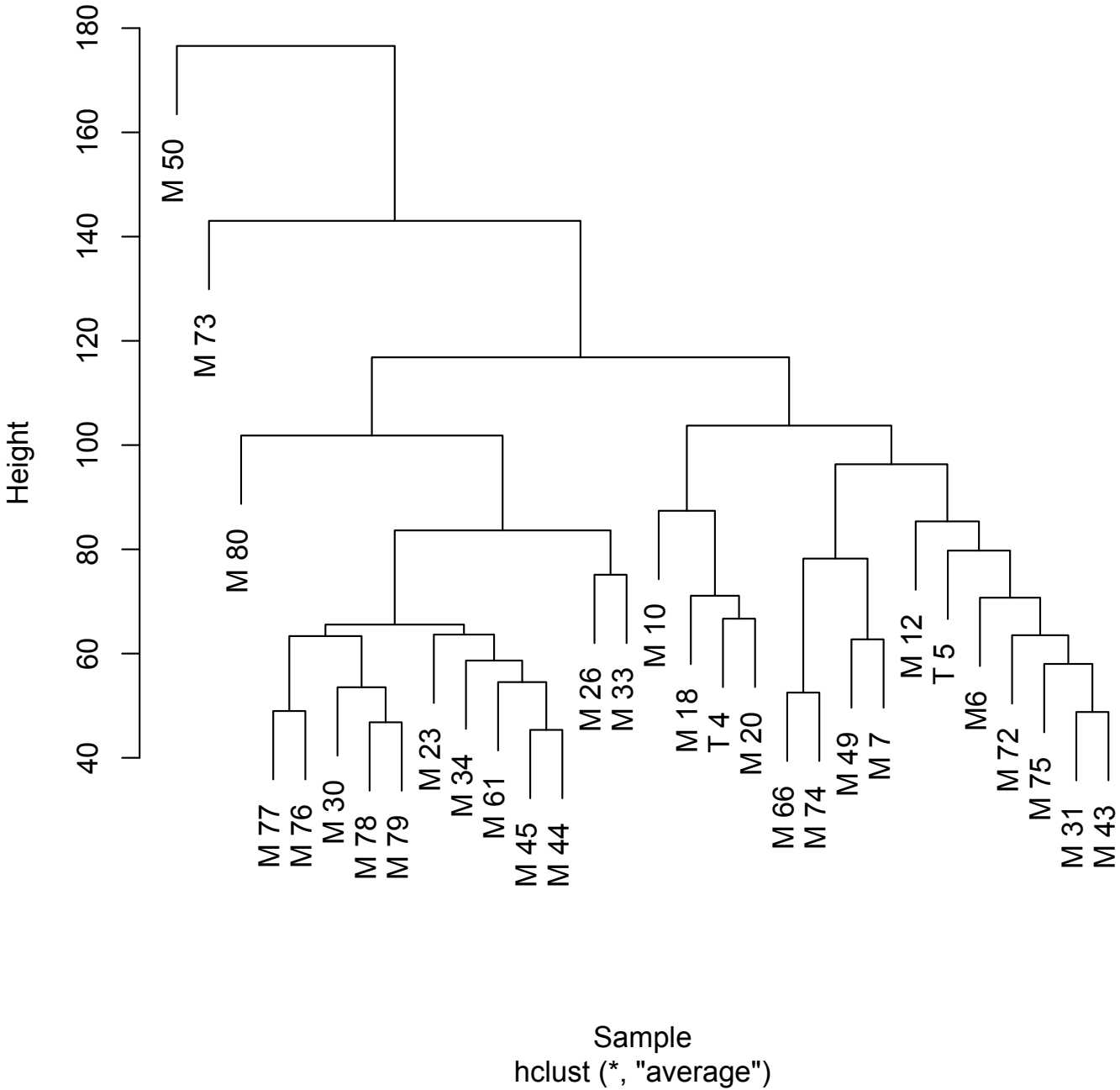

B)

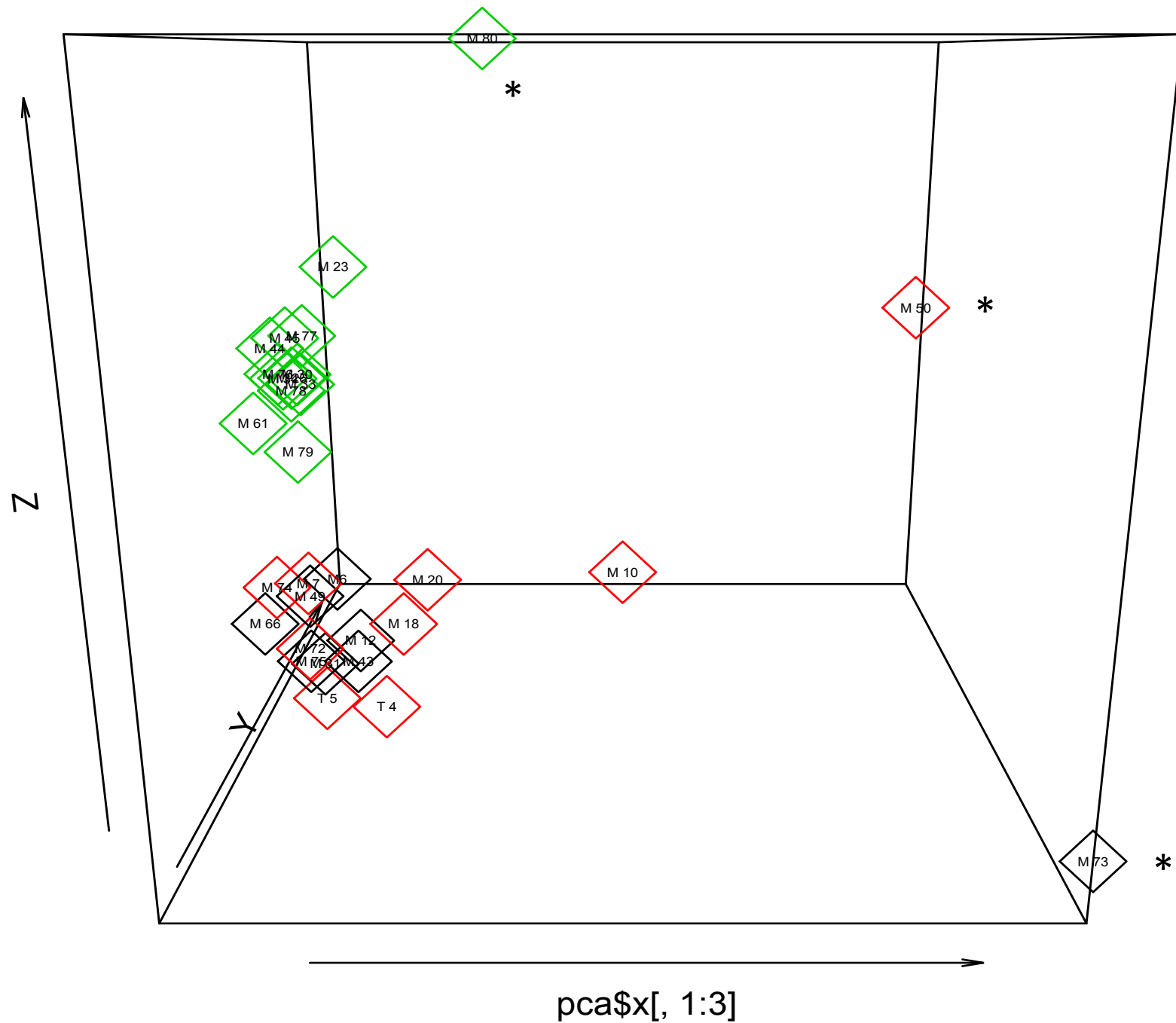

Supplement: Figure S1 — Hierarchical Clustering dendrogram (A) and Principal Components Analysis plot (B) for the raw data for all samples (i.e., all expressed probes prior to normalization) which clearly show that M80, M50, and M73 (shown with an asterix in the PCA plot) cluster away from all other samples in these plots. [file Image1.PDF]
