# Supplementary material for: Persistence of Innate Immune Pathways in Late Stage Human Bacterial and Fungal Keratitis: Results from a Comparative Transcriptome Analysis
Source: Front Cell Infect Microbiol. 2017 May 18;7:193. doi: 10.3389/fcimb.2017.00193 (PMC5435826; doi:10.3389/fcimb.2017.00193)

Supplementary Figure 2: Protein-Protein Interaction (PPI) network maps for the differentially expressed genes in 1) Bacterial Keratitis versus Control (BK v C) and 2) Fungal Keratitis versus Control (FK v C) comparisons, as well as 3) the overall network co-expression analysis map for all BK and FK differentially expressed samples.

1) BK v C PPI Network Map

The entire BK v C PPI network is shown below. MCODE analysis in Cytoscape found 6 highly interconnected clusters. The three most-highly connected clusters are shown below in red (BTK, FGR, HCK, LYN, PLCG2, KIT), green (IL8, CCR1, CXCR4, CXCL6, CCL5) and pink (IRAK2, RIPK2, IRAK3, NOD2, TLR4).

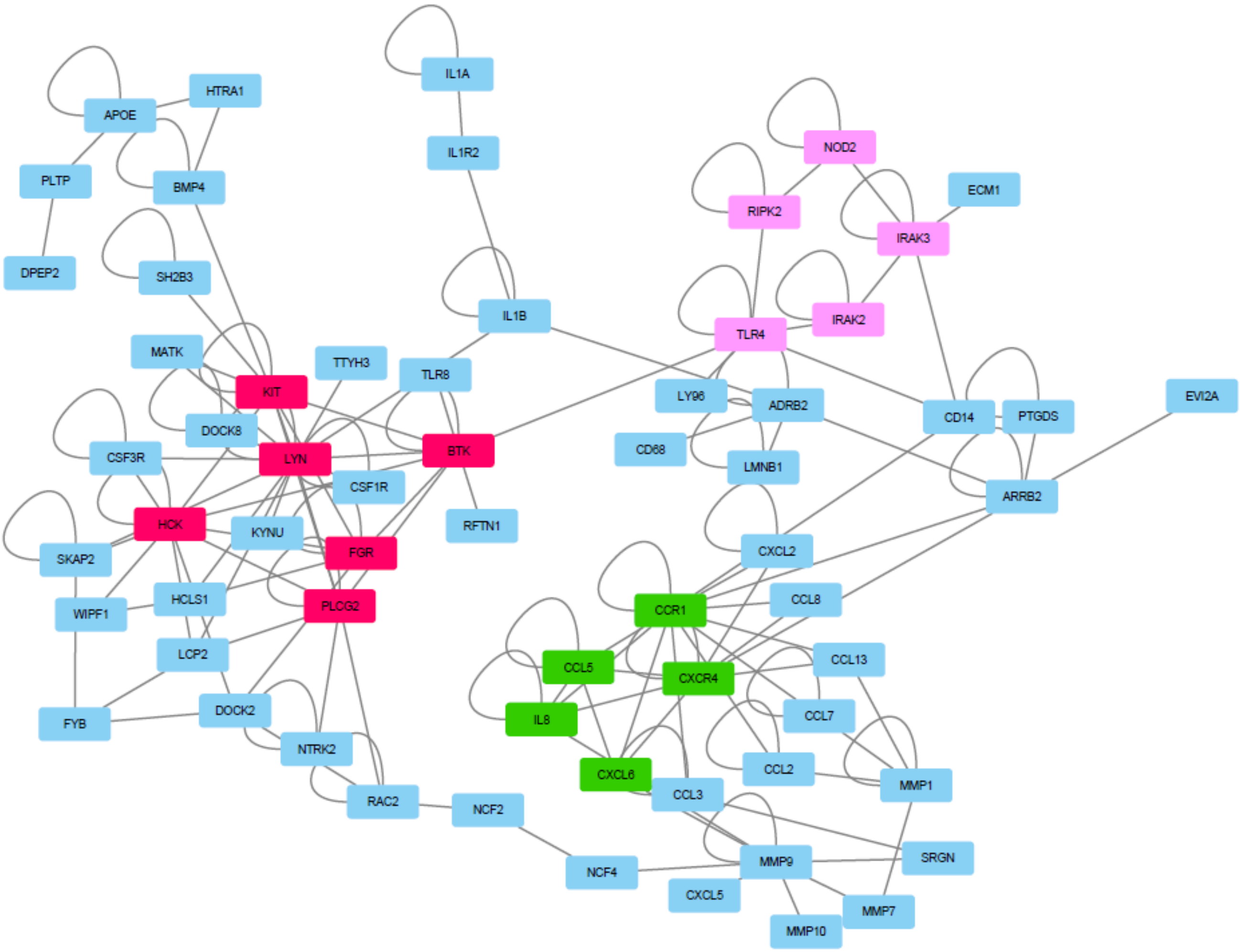

2) FK v C PPI Network Map

The entire PPI network for FK v C is shown below. MCODE analysis found only 1 highly interconnected cluster (IL8, CCL22, CXCR4, CCR1, CCL5), shown in red.

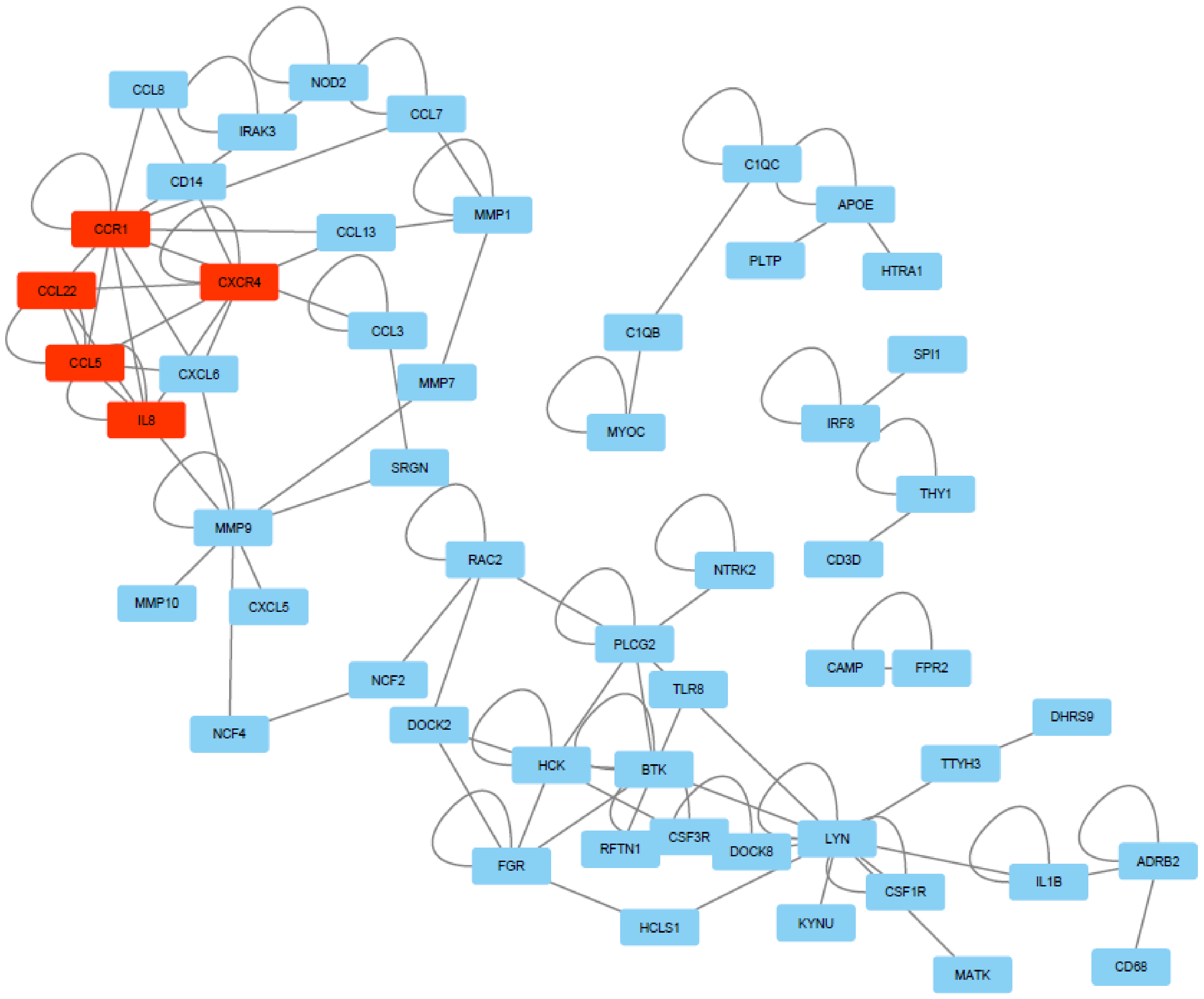

3) Network co-expression analysis map of all FK and BK differentially expressed genes. Each gene is a node, and edges were drawn to connect nodes if the Pearson correlation coefficient between the two genes was ≥0.9, using Miru. The final network has 513 nodes with 18,592 edges, forming 4 natural clusters. Using the Markov Chain clustering algorithm, there were 7 highly connected clusters (enriched GO terms in parnetheses after each cluster and further details in Table S5): cluster 1 (in red; leucocyte migration & cytokine production), cluster 2 (yellow; blood vessel morphogenesis), cluster 3 (light green; lymphoid progenitor cell and epithelial cell differentiation), cluster 4 (dark green; cytokine-mediated signaling pathway), cluster 5 (light blue; protein folding associated with cytoplasmic vesicles), cluster 6 (violet; cytoskeleton organization) and cluster 7 (navy blue; regulation of T-cell activation). Nodes shown in white represent genes that are not strongly associated with any cluster after the Markov Chain clustering algorithm had been applied.

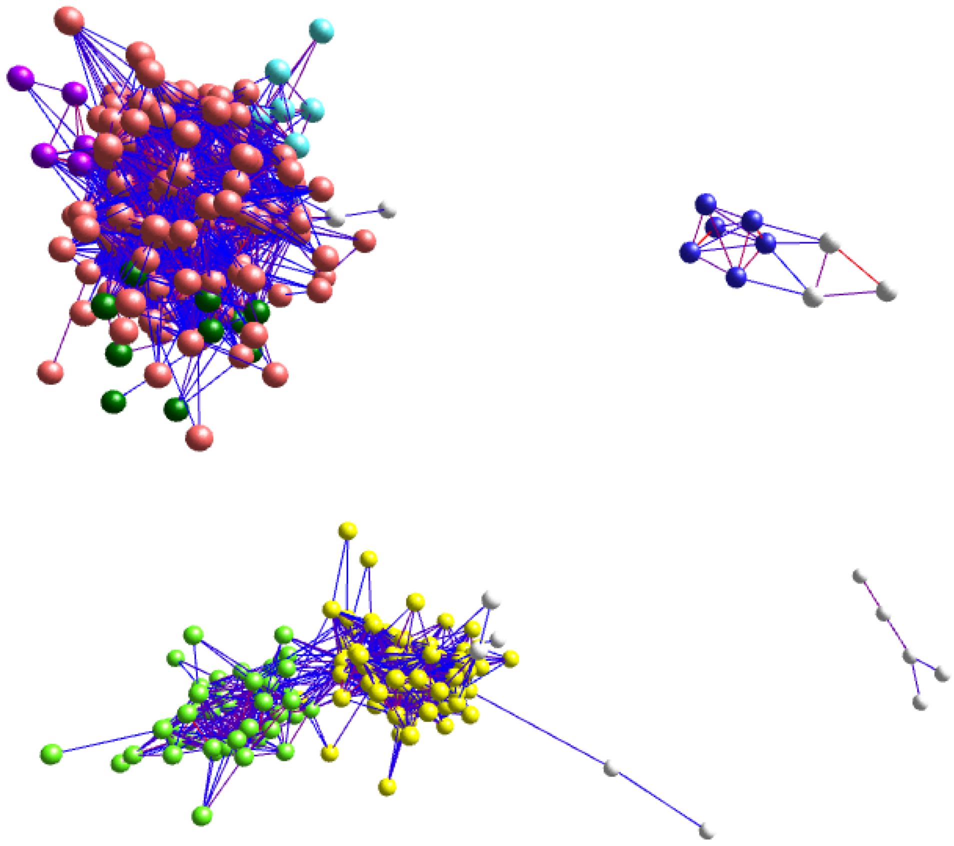

Supplement: Figure S2 — Network maps of the Protein-Protein Interaction Analysis for Bacterial Keratitis vs. Control and Fungal Keratitis vs. Control, as well as the network co-expression analysis for all differentially expressed genes in bacterial and fungal keratitis. [file Image2.PDF]
